# To investigate the effects of artemisinin on inflammatory factors and intestinal microbiota in rats with ulcerative colitis based on network pharmacology

**DOI:** 10.3389/fgstr.2022.979314

**Published:** 2022-08-12

**Authors:** Yuxi Guo, Ze Li, Nan Cheng, Xuemei Jia, Jie Wang, Hongyu Ma, Runyuan Zhao, Bolin Li, Yanru Cai, Qian Yang

**Affiliations:** ^1^ The First Affiliated Hospital of Hebei University of Traditional Chinese Medicine, Hebei University of Chinese Medicine, Shijiazhuang, China; ^2^ Department of Traditional Chinese Medicine, Hebei Provincial People’s Hospital, Shijiazhuang, China; ^3^ Department of Gastroenterology, the First Affiliated Hospital of Guangzhou University of Chinese Medicine, Guangdong, China; ^4^ Department of Gastroenterology, Hebei Provincial Hospital of Chinese Medicine, Shijiazhuang, China

**Keywords:** artemisinin, ulcerative colitis, inflammation, intestinal flora, 16s rDNA sequencing, network pharmacology

## Abstract

**Objective:**

To investigate the therapeutic effect and possible mechanism of artemisinin on ulcerative colitis (UC) induced by sodium glucan sulfate (DSS) in rats based on network pharmacology.

**Methods:**

First, according to the 3D structure of artemisinin, the effective targets of the active compounds were obtained through the Swissstarge website (www.swisstargetprediction.ch/) and the TargetNet website (http://targetnet.scbdd.com/). With the aid of Genecards (https://www.genecards.org/), OMIM (https://omim.org/), TTD (http://db.idrblab.net/ttd/) to obtain effective targets of disease. The disease gene-drug target network was constructed by extracting the intersection targets of the two, and the visualization operation and analysis were performed by using Cytoscape 3.7.2. Gene function enrichment analysis and pathway analysis were performed on the intersection targets with the help of R language software. Autidock Vina was used for molecular docking of artemisinin to key targets. Then, 40 male Wistar rats were randomly divided into normal group, model group, mesalazine group (0.315 g/kg·d) and artemisinin group (0.1 g/kg·d), with 10 rats in each group. Except for the normal group, the rats in the other groups were given 3.5% DSS solution freely for 10 days to replicate the UC model. After the successful modeling, the rats were given intragastric administration. The normal group and the model group were given the same amount of 0.9% normal saline, once a day, for 14 days. The general condition of the rats was recorded every day and the disease activity index (DAI) score was performed. After the administration, the colonic mucosal damage index (CMDI) was scored, the histopathological changes of the colon were observed by HE staining, and the levels or activities of serum CRP, TNF-α, MDA, SOD, HIF-1α and T-AOC were detected by ELISA, and fecal and intestinal microbiota of rats were detected by 16S rDNA sequencing.

**Results:**

Network pharmacology shows that, there were 98 key targets of artemisinin screening, 4853 effective targets of UC, and 43 intersection targets for artemisinin and UC, involving 48 signaling pathways. The molecular docking results showed that the binding energies of the key proteins to artemisinin were less than -5.0 kJ·mol^-1^, and the binding energy of PTGS2 NOS3 to artemisinin was the best. Animal experiments have shown that, Compared with the model group, the DAI and CMDI scores of the artemisinin group and the mesalazine group decreased, the levels and activities of serum CRP, TNF-α, MDA and HIF-1α decreased, the levels and activities of SOD and T-AOC increased, the abundance and diversity of inteatinal microbiota increased, and the abundance of p-Acidobacteria, p-Chloroflexi, p-Gemmatimonadetes, p-Nitrospirae in artemisinin group increased (*P＜*0.05), and there was no significant change in others.

**Conclusion:**

Artemisinin intervenes with UC through key target proteins such as PTGS2 and ESR1, and involves various biological processes such as inflammation and intestinal microbiota, revealing that molecular basis of artemisinin in the treatment of UC. Artemisinin is effective in improving the symptoms of UC rats, and its mechanism may be to relieve oxidative stress response by inhibiting inflammation, thus promoting intestinal mucosal repair. The regulatory effect on intestinal microbiota needs to be further studied.

## 1 Introduction

Ulcerative colitis (UC) is a chronic non-specific intestinal inflammatory disease, which mainly accumulaes the mucosa and submucosa of the rectum and colon. The typical clinical manifestations are abdominal pain, acute and severe diarrhea accompanied by mucus, pus, blood and stool, etc. Studies have found that the pathogenesis of UC is closely related to genetic susceptibility to intestinal microbiota disorder, immune disorders, intestinal mucosal barrier damage, oxidative stress and other factors. Among them, intestinal microbiota, inflammatory factors and oxidative stress played an important role in the pathogenesis of UC and has received extensive attention in recent years (1, 2). Intestinal immune dysfunction and the imbalance of inflammatory factors are considered to be the direct causes of UC (3), which are mainly manifested in the imbalance of pro-inflammatory and anti-inflammatory factors. The species richness, diversity and stability of the intestinal microbiota are also closely related to the activity of intestinal diseases, and the composition of the intestinal microbiota in UC patients is significantly different from that in normal people (4–8). For example, the expression content of Escherichia coli, Streptococcus, and Bacillus difficile increased, while the expression of Bifidobacterium, Lactobacillus, Clostridium and other probiotics decreased (9), which destroyed the intestinal mucus layer and further aggravated the inflammatory response (10). At the same time of UC inflammation, due to the infiltration of a large number of neutrophils, the oxygen consumption increased during phagocytosis, and a large number of oxygen free radicals are generated, which induces oxidative stress reaction in the body. The two promote each other and destroy the intestinal mucosa together, which cause tissue damage and aggravate the disease.

At present, western medicine mainly focuses on anti-inflammatory and immune regulation, supplemented by biological agents. Related clinical drugs, such as mesalazine, prednisone, azathioprine, infliximab, etc. have obvious side effects while providing therapeutic effects. They are prone to relapse after withdrawal, and their prices are high, with great potential risks, which need to be further studied and discussed. However, Traditional Chinese medicine has gained some attention in the treatment of UC due to its advantages of multi-target and multi-link regulation.

Artemisinin is an effective ingredient extracted from the Chinese herbal medicine Artemisia annua. It has a wide range of pharmacological effects, which are basically the same as those of traditional Chinese herbal medicine, such as anti-malaria, anti-bacteria and anti-insect, anti-inflammation, anti-oxidation, anti-tumor, anti-pyretic, immunomodulatory and other effects. In recent years, related researches have been carried out gradually (11–15), but the treatment and mechanism of UC are still not perfect, mostly from the perspective of immune regulation, lack of analysis of intestinal microbiota. Therefore, this study attempted to further explore the therapeutic effect of artemisinin on UC and the related mechanism by observing the changes of inflammatory factors and oxidative stress indexes of intestinal microbiota in rats after artemisinin treatment

## 2 Materials and methods

### 2.1 Materials and reagents

Artemisinin (Tixiai (Shanghai) Chemical Industry Development Co., Ltd., CAS RN: 63968-64-9, product code: A2118, purity>97.0%); mesalazine enteric-coated tablets (Salfo), Production batch number: L19050A, Imported drug registration number: H20171358, Specification: 0.5g × 40 tablets.

Dextran Sodium Sulfate (DSS) (Dalian Meilun Biotechnology Co., Ltd., Product No. 00806A); Enzyme-linked immunosorbent assay (ELISA) kits for TNF-α、MDA and SOD were obtained from Jiangsu Meimian inductrial Co., Ltd. (Yancheng, Jiangsu, China). Enzyme-linked immunosorbent assay (ELISA) kits for CRP、T-AOC and HIF-1α were obtained from Nanjing Jiancheng Institute of Bioengineering.

### 2.2 Animals and experimental design methods

A total of 40 SPF male Wistar rats (age, 8-week-old; weighing, 200± 20g) were provided by the Animal Experiment Center of Hebei Medical University [SCXK (Hebei) 2018- 004].The breeding environment was room temperature 20-25℃, humidity 40%-60%. The rats were fed for 7 days, during which water and food were freely consumed, and pads were changed regularly.

Forty male Wistar rats were divided into blank group of 10(N) and model group of 30 according to random number table method. Among them, the modeling group freely drank DSS solution (dissolving DSS in distilled water to prepare a fresh solution with a concentration of 3.5% daily), and the blank group drank distilled water. After 10 days, 3 rats were randomly selected from the modeling group, sacrificed, and their colon tissues were observed. Obvious pathological changes such as hyperemia, edema, erosion, and ulceration were observed with the naked eye, that is, the modeling was judged to be successful. Rats with successful modeling were divided into model group (M), mesalazine group (Western medicine, W) and artemisinin group (A) according to the random number table method, with 9 rats in each group.

The doses of drugs administered to rats by intragastric administration were calculated by the body surface area conversion algorithm between humans and rats, and the blank group and model group were intragastrically administered with equal doses of normal saline. After calculation, mesalazine group was given mesalazine suspension 315 mg/kg·d, artemisinin group was given artemisinin suspension 0.1 g/kg·d, each group was given once a day, continuous intragastric administration for 2 weeks. During the treatment, one rat died in each of the model group, mesalazine group and artemisinin group, and it was found that they might have died of toxic megacolon obstruction through anatomical and histological observation.

### 2.3 DAI score

The hair gloss, mental state, activity, water intake and food intake of the rats were observed regularly every day. If necessary, the rats were stimulated to defecate by massage. The color, texture and smell of the feces were observed, and fecal occult blood was detected. The above conditions were evaluated according to the DAI scoring standard established by Murano et al. (16) (**Table 1**). DAI= (weight loss score + stool trait score + blood in the stool score)/3. The scoring scale ranges from 0 to 4, and the higher the score, the higher the severity of inflammation.

**Table 1:** DAI scoring standard of rats.

Score	Weight Loss(%)	Stool consistency	Occult/gross bleeding
0	None	Normal	Negative
1	1-5	–	–
2	5-10	Loose stools	Hemoccult positive
3	10-15	–	–
4	>15	Diarrhea	Gross bleeding

### 2.4 Rat CMDI (colon mucosal injury index) score

The rat colon was cut along the frenulum, washed with normal saline, and then spread on filter paper, and the residual water on the surface was blotted dry. The colonic mucosal congestion and edema, erosions, ulcers, and bleeding spots were observed, and the colonic mucosal injury index was scored (**Table 2**) (17).

**Table 2:** The standard of CMDI score.

Score	Visual inspection of colon tissue
0	The mucosa is intact without damage.
1	Mild congestion, edema, smooth surface, no erosion or ulcer on mucosal surface.
2	Moderate congestion, edema, and erosions on the mucosal surface.
3	Severe congestion, ulceration or even necrosis on the mucosal surface, the main ulcer area is less than 40%.
4	Severe congestion, ulceration or even necrosis on the mucosal surface, the main ulcer area is ≥40%.

### 2.5 Colon histopathology

Colon tissue fixed with 4% paraformaldehyde was taken out, routinely dehydrated, embedded in paraffin, sectioned, stained with HE, and observed under light microscope for pathological changes related to colonic mucosa, such as lamina propria vascular lesions, glandular lesions, inflammatory cells Infiltration, interstitial changes and crypt abscess.

### 2.6 Network pharmacology analysis

According to the 3D structure of artemisinin, combined with database collection and literature search, the effective targets of artemisinin compounds were predicted.

The effective targets of active compounds were obtained *via* swisstage website (www.swisstargetprediction.Ch/) and targetnet website(http://targetnet.scbdd.com/).

Using genecards(https://www.genecards.org/),OMIM(https://omim.org/), TTD (http://db.idrblab.net/ttd/)to gain effective targets of diseases. Finally, the disease gene drug target network is constructed by extracting their intersection targets, and the visualization operation is carried out by Cytoscape 3.7.2.

### 2.7 Gene ontology analysis and pathway analysis and molecular docking verification

In order to further study the molecular mechanism of artemisinin in the treatment of UC, the gene function enrichment analysis and pathway analysis (*P＜*0.05) of the intersection targets were performed with the help of R language software, and visualization operations were performed.

The PDB database (https://www.rcsb.org/) was used to download the protein structures of 10 key targets. AutoDock Vina software was used for molecular docking of artemisinin with 10 key target proteins, and Pymol was used to draw the optimal docking results.

### 2.8 ELISA results

ELISA was used to detect the content or activity of CRP, TNF-α, MDA, SOD, HIF-1α and T-AOC in serum, and the operations were carried out in strict accordance with the kit instructions.

### 2.9 16S rDNA sequencing and intestinal microbiota analysis

The total DNA of fecal bacteria was extracted from the rats in each group according to the operation steps of the FastDNA Spin Kit (Mobio, Carlsbad, USA), and the DNA concentration was measured with Qubit^®^ 2.0 fluorescent agent (Life Technology, Carlsbad, CA, USA). The variable region of 16S rRNA gene V4 was amplified by PCR, and the primer sequences were 515F: 5’-CCTAYGGGRBGCASCAG-3’; 806R:5’-GGACTACNNGGGTATTCTAAT-3’. Illumina NovaSeq PE250 platform for 16S rDNA sequencing: 2200 TapeStation (Agilent) for purification and quantification, clustering of valid sequences into taxonomic units (OTUs), species annotation for OUTs, and species distribution histograms at the phylum and genus levels.

The Alpha diversity index (including Chao1, Ace, Simpson, Shannon, and Goods Coverage index) of colonic microorganisms was calculated by Mothur software, and the microbiota abundance and diversity were evaluated. R software (Version 2.15.3) was used to draw PCA, PCoA and NMDS graphs, and finally, the statistical differences of Biomarker between groups were analyzed by LefSe (Line Discriminant Analysis (LDA) Effect Size).

### 2.10 Statistical analysis

Data were analyzed using SPSS 26.0 and Graphpad Prism 7 statistical software packages, the statistical data were expressed as $\bar{x}\pm s$ , and the comparison between groups was performed by one-way ANOVA, with *P＜*0.05 indicating that the difference was statistically significant.

## 3 Results

### 3.1 DAI in DSS-induced UC rats

**Figure 1:**
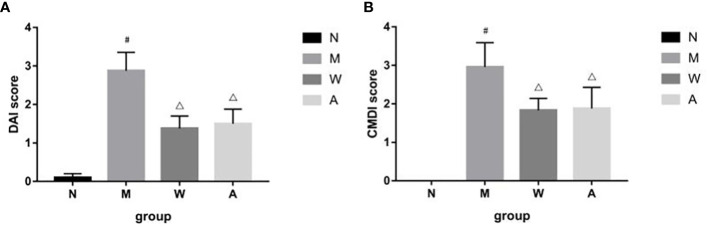
**(A)** DAI score in DSS-induced UC rats. **(B)** CMDI score in DSS-induced UC rats. N, normal group; M, rats treated with 3.5% DSS alone; W: rats treated with DSS plus mesalazine (315 mg/kg); A, rats treated with DSS plus Artemisinin (100 mg/kg). ^#^ *P＜* 0.05 *vs* N.^△^ *P＜*0.05 *vs* M.

The rats in the normal group had the weight at a constant rate, shiny hair, lively spirits, agile movements, quick reactions, normal diet and normal urine and faeces. The model group began to lose weight gradually since the modeling, recumbent and lazy movement, hair thinning and loss of luster, poor spirit, slow movement, slow reaction, anorexia, drinking less, perianal filth, stool gradually from sparse and frequent to mucus, pus and blood stool. Rats in the mesalazine group and artemisinin group all improved after treatment, the activities increased compared with that during the modeling period, hair was gradually plump but still dull, mental state and diet basically recuperated, and the perianal area was gradually cleaned. Formed stool began to appear on the 5th day of drug treatment, and the mucus, pus and blood gradually disappeared. Compared with the normal group (0.10 ± 0.10), the DAI score of the rats was higher in the model group (2.88 ± 0.48), and the difference was statistically significant (*P＜*0.05); Compared with model group, the scores of mesalazine group (1.38 ± 0.32) and artemisinin group (1.11 ± 0.35) were decreased, and the differences were statistically significant (*P＜*0.05). There was no significant difference between mesalazine group and artemisinin group (*P＞*0.05). (**Figure 1**).

### 3.2 CMDI in DSS-induced UC rats

After the experiment, the rat colon tissue was collected and observed. Macroscopically, the colon surface of the rats in the normal group was smooth, pale pink, with clear texture, no congestion and edema, and no erosion and ulcer; the rats in the model group had swollen intestines, and the surface of the colon mucosa was rough and fine-grained, dark red, with obvious congestion, edema, and erosion, the ulcer surface is larger, and some intestinal tubes are adhered to other organs and abdominal wall, and even necrosis occurs; the intestinal tubes of rats in the mesalazine group are slightly swollen, the surface of the colon mucosa is not smooth, the folds are arranged neatly, light red, and mild hyperemia and edema, no obvious ulcer surface, occasionally scattered spots and erosions, and occasional adhesions between the intestinal tube and surrounding tissues; the intestinal tube of the rats in the artemisinin group was slightly swollen, the surface of the colonic mucosa was smooth, the folds were arranged regularly, light red, and slightly congested Edema, no necrosis, no adhesion to surrounding tissue, slight congestion and edema, no obvious erosion and ulcer. Compared with the normal group (0), the CMDI score of the rats in the model group (2.96 ± 0.63) increased, and the difference was statistically significant (*P＜*0.05); Compared with the model group, the score of the mesalazine group (1.83 ± 0.31) and the artemisinin group (1.03 ± 0.86) decreased, and the difference was statistically significant (*P＜*0.05). There was no significant difference between mesalazine group and artemisinin group (*P＞*0.05) (**Figure 1**).

### 3.3 Histopathology in DSS-induced UC rats

**Figure 2:**
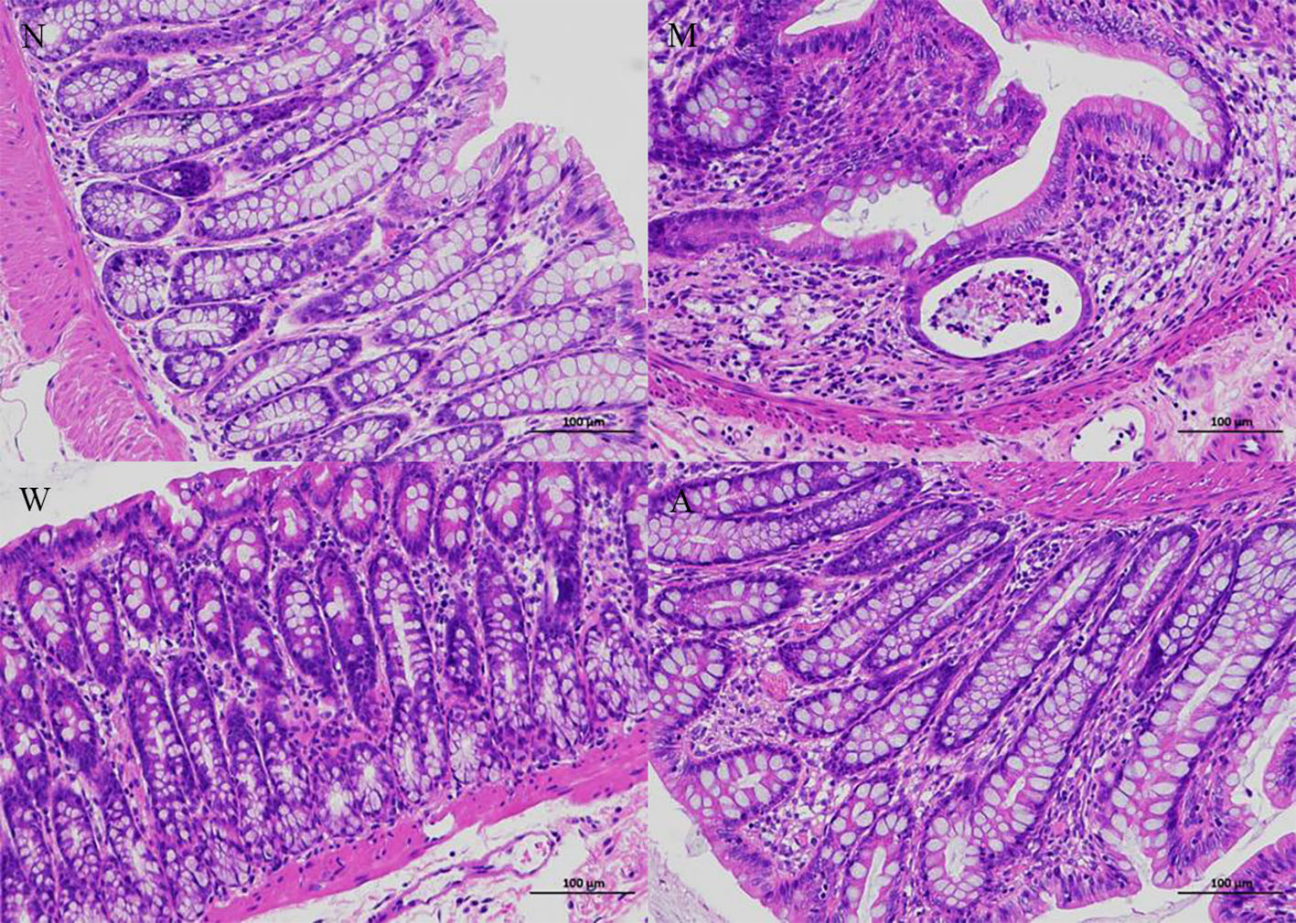
Histopathology (magnifification 200×). N, normal group; M, rats treated with 3.5% DSS alone; W, rats treated with DSS plus mesalazine (315 mg/kg); A, rats treated with DSS plus Artemisinin (100 mg/kg).

As shown in **Figure 2**, the colonic tissue was stained with HE staining and observed under a microscope. The colonic mucosa of the rats in the normal group was intact, the cells were neatly arranged, the glandular structure of the lamina propria was normal, and there was no infiltration of inflammatory cells. Instead of normal cells, goblet cells decreased, the crypt structure was disordered, crypt abscesses appeared, and glands atrophied; the structure of the colonic mucosa of rats in the mesalazine group was basically restored, the cells were arranged regularly, the gland structure was relatively complete, some parts were still visible, and some inflammatory cells infiltration can still be seen; the colonic mucosa of the rats in the artemisinin group was relatively intact, the cells were arranged regularly, the intestinal gland structure returned to normal, and a small amount of inflammatory cells infiltration ware seen.

### 3.4 Network pharmacology analysis and molecular docking results of artemisinin

**Figure 3:**
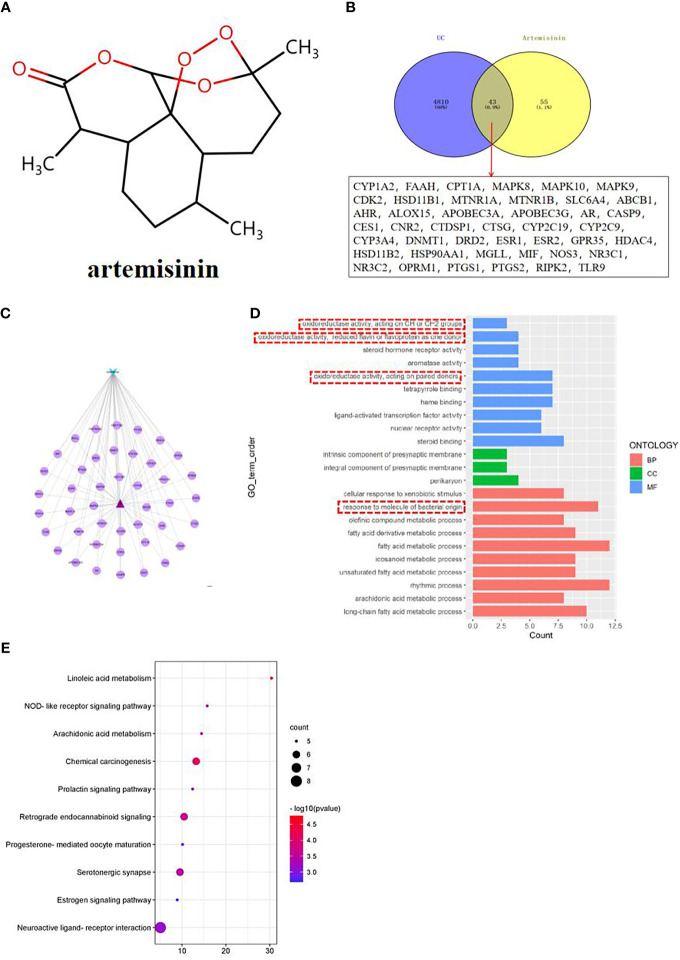
**(A)** Network pharmacological analysis of artemisinin against UC; **(B)** Target Venny analysis chart of artemisinin against UC; **(C)** Protein interaction network of artemisinin against UC; **(D)** GO enrichment analysis of intersection targets of artemisinin in treating UC; **(E)** Top 10 channel bubble map of intersection targets of artemisinin in treating UC by KEGG enrichment analysis.

According to the 3D structure of artemisinin (**Figure 3A**), the targets were obtained, and the intersection targets of UC and artemisinin (**Figure 3B**) were gained. And then the “disease gene drug target” network (**Figure 3C**) was established through Cytoscape 3.7.2, including 43 149 nodes and 149 edges, with an average degree of 7.95.The top 10 key targets in degree value are: PTGS2 (prostaglandin endoperoxide synthase 2), ESR1 (estrogen receptor), HSP90AA1 (No. 14 Chromosomal genes), NR3C1 (genes on chromosome 5), MAPK8 (mitogen-activated protein kinase 8), CYP3A4 (cytochrome P4503A4 enzyme), AHR (aromatic hydrocarbon receptor), ABCB1 (P-glycoprotein), AR (androgen receptor). These results suggest that the above targets may play an important role in the process of artemisinin’s intervention in UC. Numerous studies have shown that inhibition of PTGS2 content reduces inflammation and the severity of experimental UC, confirming its critical role in the development of UC (18, 19). Furthermore, COX-2-PGE2 signaling impairs intestinal epithelial regeneration and correlates with responsiveness to TNF inhibitors in UC (20, 21). It further suggests

the close connection between UC and inflammatory response, so our research group measured the related indicators of inflammatory response.

The results of GO enrichment analysis of the two intersection targets showed that the biological processes of artemisinin in the treatment of UC mainly involved biological processes such as fatty acid metabolic process, response to molecule of bacterial origin, rhythmic process, etc., cellular components such as intrinsic components of the presynaptic membrane and perikaryon, molecular functions such as oxidoreductase activity, steroid hormone receptor activity, etc (**Figure 3D**). Among them, the response to molecule of bacterial origin relatively ranks high, suggests that regulating the intestinal microbiota can help treat UC. Observations from previous clinical and experimental studies suggested that intestinal bacterial imbalance is associated with disease initiation and progression in UC (22, 23). Therefore, this topic will be discussed in detail from the regulation of intestinal microbiota in the treatment of UC. The results of KEGG analysis indicated that the process of artemisinin’s intervention in UC was closely related to metabolism, apoptosis, endocrine, nerve conduction, and immune response-related pathways (**Figure 3E**), which also provided new directions and ideas for our next research.

**Figure 4:**
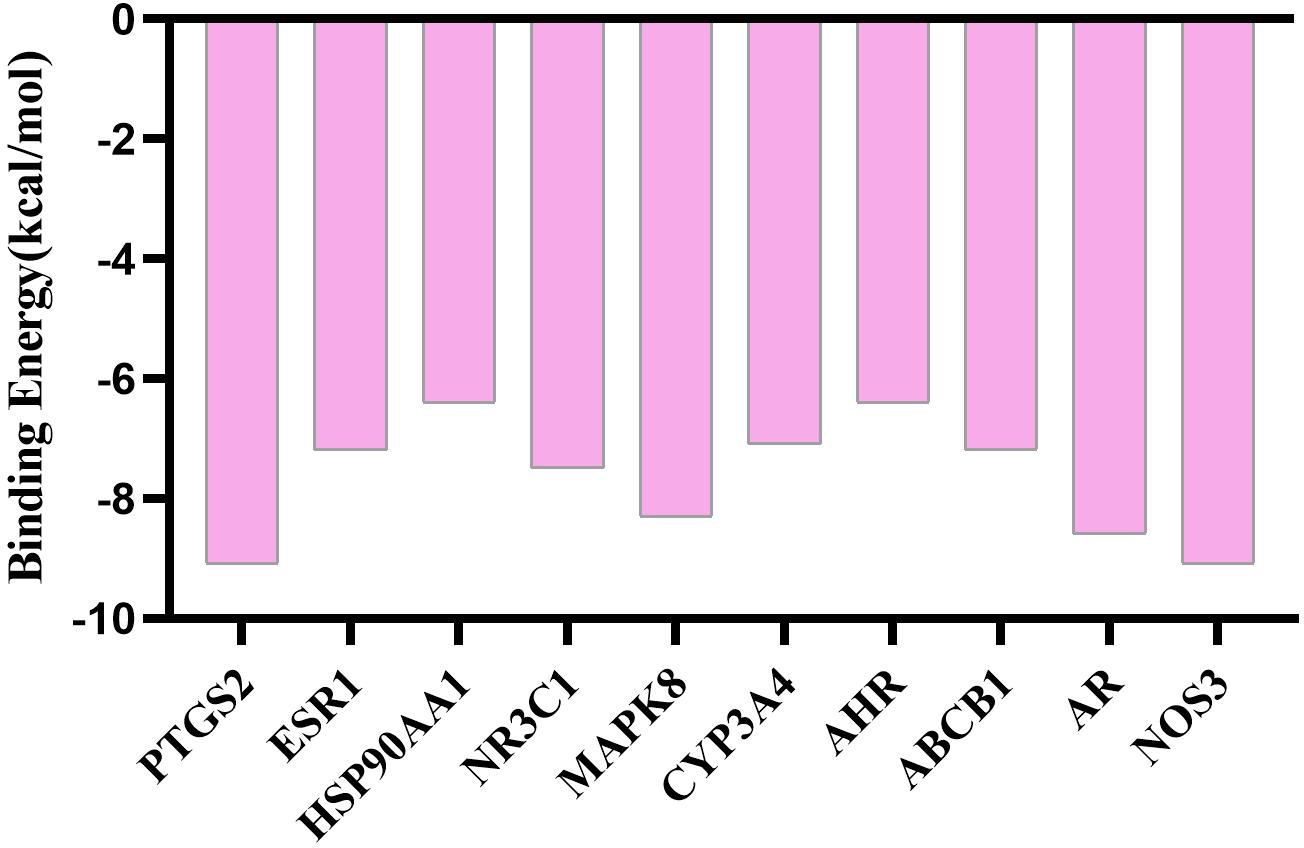
Histogram of docking results of artemisinin with 10 core target protein molecules.

**Figure 5:**
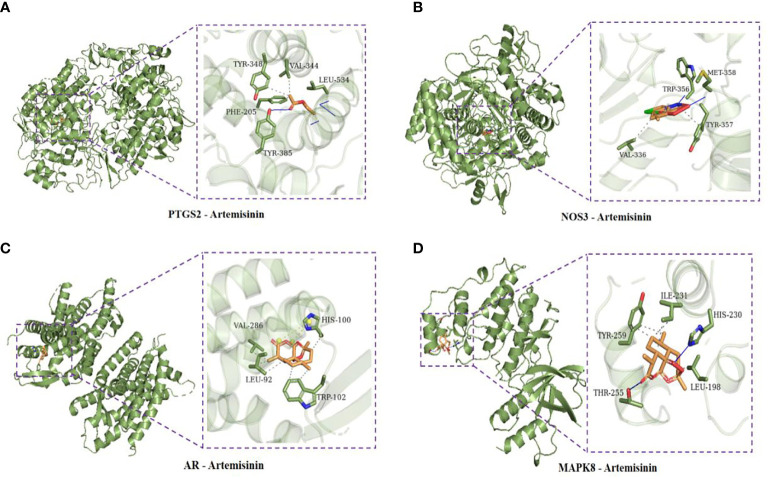
**(A)** Docking conformation of PTGS2 and artemisinin. **(B)** Docked conformation of NOS3 and artemisinin. **(C)** Docked conformation of AR and artemisinin. **(D)** Docked conformation of MAPK8 and artemisinin.

Using AutoDock Vina software, the top 10 core target genes were molecularly docked with artemisinin to verify its efficacy. The docking results (**Figure 4**) showed that the binding energies of the 10 results were relatively good (Total score ≤ - 5.0), and the 4 groups with the best docking situations were plotted (**Figure 5**), suggesting that artemisinin may intervene in UC through key targets such as PTGS2, ESR1, HSP90AA1, and NR3C1.

### 3.5 Effects of CRP, TNF-α, T-AOC, HIF-1α, MDA, SOD levels in serum of UC rats

**Figure 6:**
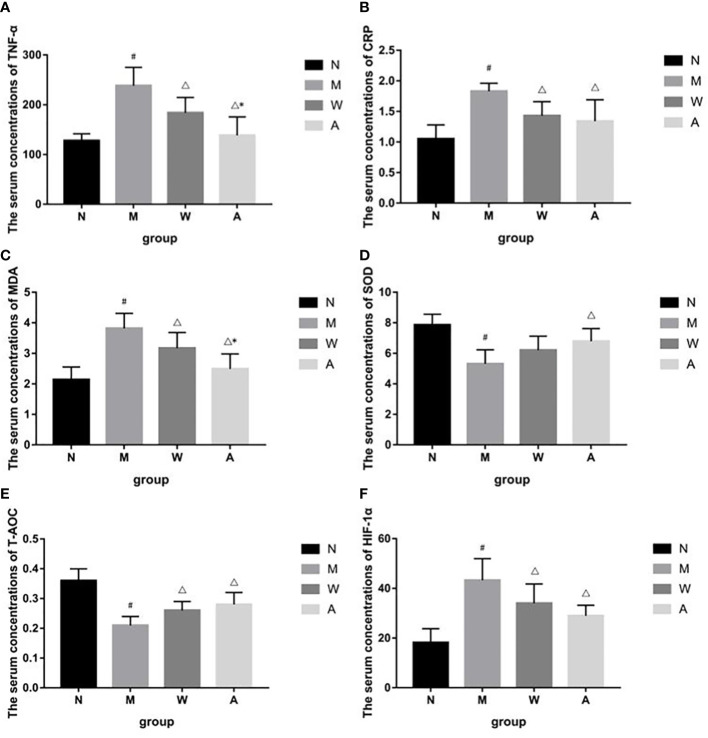
**(A)** Effects of TNF-á levels in serum of UC rats. **(B)** Effects of CRP levels in serum of UC rats. **(C)** Effects of MDA levels in serum of UC rats. **(D)** Effects of SOD levels in serum of UC rats. **(E)** Effects of T-AOC levels in serum of UC rats. **(F)** Effects of HIF-1á levels in serum of UC rats. N, Normal rats; M, rats treated with 3.5% DSS alone; W, rats treated with DSS plus Mesalazine (315 mg/kg); A, rats treated with DSS plus Artemisinin (100 mg/kg).^#^ *P＜*0.05 *vs* N.^△^ *P＜*0.05 *vs* M.^*^ *P＜*0.05 *vs* W.

Compared with the normal group, the serum levels of TNF-α and CRP in the model group were significantly increased (*P*<0.05); compared with the model group, the serum TNF-α and CRP levels in the mesalazine group were significantly decreased (*P＜*0.05); compared with the mesalazine group, the serum TNF-α content of the artemisinin group was significantly decreased (*P＜*0.05), and there was no significant difference in the CRP content (*P＞*0.05) (**Figures 6A, B**).

Compared with the normal group, the serum HIF-1α, MDA content or activity of the rats in the model group was significantly increased (*P＜*0.05); compared with the model group, the serum HIF-1α, MDA content or activity of the rats in each drug group was significantly decreased (*P＜*0.05); compared with the mesalazine group, the serum MDA activity of the artemisinin group was decreased (*P＜*0.05), and there was no significant difference in the content of HIF-1α (*P＞*0.05)(**Figures 6C, F**).

Compared with the normal group, the serum T-AOC and SOD contents of the model group were significantly decreased (*P＜*0.05); compared with the model group, the serum T-AOC and SOD contents of the artemisinin group were significantly increased (*P＜*0.05), the serum T-AOC content of the mesalazine group was significantly increased (*P＜*0.05), and the SOD content had no significant difference (*P＞*0.05).There was no significant difference between mesalazine group and artemisinin group (*P＞*0.05) (**Figures 6D, E**).

### 3.6 Effects of artemisinin on intestinal microbiota of UC rats

#### 3.6.1 Basic quality control analysis

**Figure 7:**
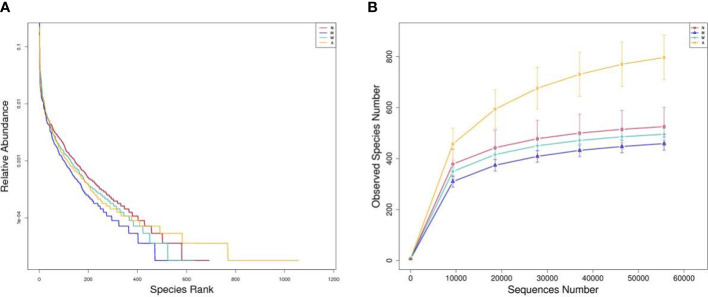
**(A)** Rank-abundance graph. **(B)** Observed species number graph. N, normal group; M, rats treated with 3.5% DSS alone; W, rats treated with DSS plus mesalazine (315 mg/kg); A rats treated with DSS plus Artemisinin (100 mg/kg).

As shown in **Figures 7A, B**, the Shannon and Rank curves of each group are relatively flat and concentrated, indicating that the amount of sequencing data is sufficient and can reflect most of the microbial information in the samples.

#### 3.6.2 Alpha diversity analysis

Alpha diversity index can reflect the richness and diversity of rat intestinal microbiota, including Chao, Ace, Simpson and Shannon indices, in which Chao and Ace reflect species abundance. The larger the values, the higher the species richness; Simpson and Shannon reflect community diversity, the smaller the Simpson index and the larger the Shannon index, the higher the species diversity. The results showed that, compared with the normal group, the Simpson and Shannon indexes of the model group were decreased, and the difference was statistically significant (*P＜*0.05), and they both rebounded after the medication, and the difference was statistically significant (*P＜*0.05); the Chao and Ace indexes of the artemisinin group were significantly higher than the model group and mesalazine group (*P＜*0.01). The above data indicate that artemisinin can improve the richness and diversity of intestinal microbiota in UC rats. The coverage value of each group was greater than 0.9, indicating that the sample detection coverage was complete (**Table 3**).

**Table 3:** Comparison of intestinal microbial diversity of rats in each group.

Group	Chao1	Ace	Simpson	Shannon	Goods average
N	568.53 ± 85.49	568.7285.39	0.93 ± 0.48	5.97 ± 0.81	0.999 ± 0.000
M	495.02 ± 27.89	501.9328.51	0.84 ± 0.11^#^	4.74 ± 0.75^##^	0.999 ± 0.000
W	528.24 ± 26.77	529.9728.76	0.92 ± 0.06^*^	5.60 ± 0.67^*^	0.999 ± 0.000
A	974.63 ± 121.46^**△△^	988.36116.73^**△△^	0.92 ± 0.40^*^	5.61 ± 0.54^*^	0.997 ± 0.000

Results are expressed as mean ± SD. ^#^P<0.05 and ^##^P<0.01 vs N; ^△△^P<0.01 vs M;^*^P<0.05 and ^**^P<0.01 vs W.N: normal group; M:rats treated with 3.5% DSS alone; W: rats treated with DSS plus mesalazine (315 mg/kg);A: rats treated with DSS plus Artemisinin (100 mg/kg).

#### 3.6.3 OTU cluster analysis

**Figure 8:**
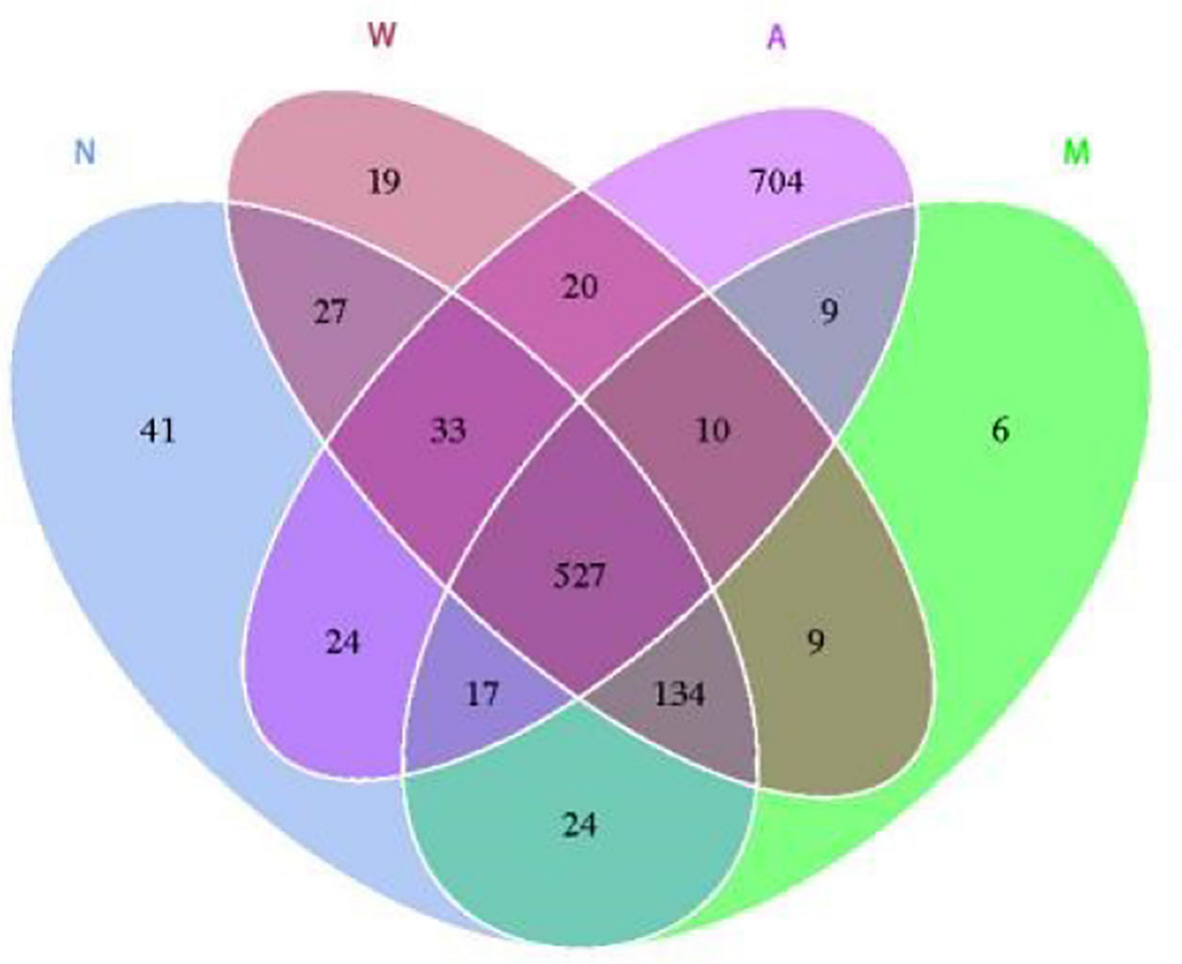
Venn diagram indicated the differential numbers of OTUs in Normal, Model, Mesalazine and Artemisinin group. N, normal group; M, rats treated with 3.5% DSS alone; W, rats treated with DSS plus mesalazine (315 mg/kg); A, rats treated with DSS plus Artemisinin (100 mg/kg).

As shown in **Figure 8**, the results of OTU cluster analysis showed that: the normal group was 827, the model group was 736, the mesalazine group was 779, and the artemisinin group was 1344, indicating that the model rats treated with artemisinin can improve the diversity of intestinal microbiota species. There are 527 identical OTUs in four groups. The normal group had 702 OTUs identical with the model group and 601 otUs identical with the artemisinin group. There were 563 OTUs in model group and artemisinin group.

#### 3.6.4 Analysis of the structure of intestinal microbiota of rats in each group

##### 3.6.4.1 Composition of the four groups of microbiota at the phylum level

**Figure 9:**
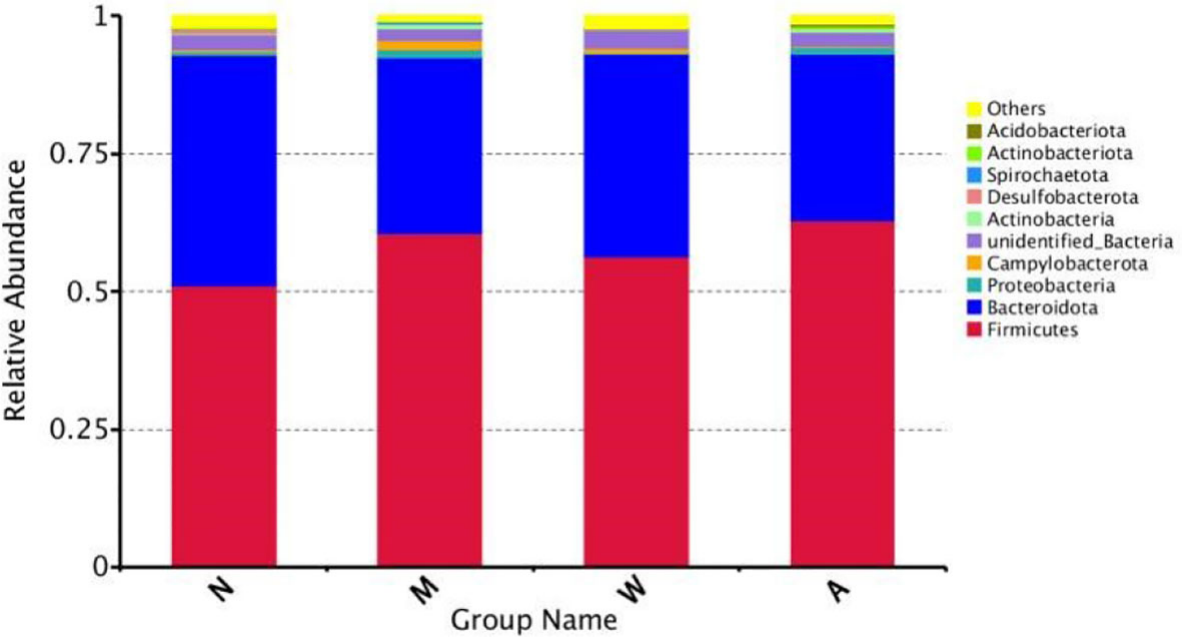
Composition of the Four Groups of microbiota at the Phylum Level. N, normal group; M, rats treated with 3.5% DSS alone; W, rats treated with DSS plus mesalazine (315 mg/kg); A, rats treated with DSS plus Artemisinin (100 mg/kg).

As shown in **Figure 9**, at the phylum level, the dominant microbiota of the four groups of samples were Firmicutes, Bacteroidetes and Proteobacteria, and the proportions of the normal group were 55.80%, 41.51% and 1.69%, respectively; the proportions of the model group were 64.09%, 31.27% and 1.69%, respectively. 1.74%; mesalazine group accounted for 61.48%, 36.31% and 1.12%; artemisinin group accounted for 65.05%, 30.32% and 2.02%. At the phylum level, each group of samples showed obvious individual differences: Firmicutes accounted for 35.57% to 80.58% of the samples, and Bacteroidetes accounted for 9.83% to 63.41% of the samples. The proportion of Proteobacteria in each sample ranged from 0.37% to 5.57%.

##### 3.6.4.2 Composition of four groups of microbiota below the phylum level

At the class level, the dominant microbiota of the 4 groups of samples were Clostridium and Bacillus of Firmicutes, followed by Bacteroidetes of Bacteroidetes; at the order level, the dominant microbiota was Bacteroidetes of Bacteroidetes, Lactobacilli of the phylum Acidobacteria, Clostridium and Erysipelotrichales of the phylum Firmicutes; at the family level, the dominant microbiota comes from the phylum Prevotaceae, Lachnospira and Ruminococci of the phylum Firmicutes, and the phylum Bacteroidetes of the Bacteroidetes family, the Lactobacillus family of the phylum Acidobacteriaceae.

#### 3.6.5 Analysis of differences in bacterial microbiota composition

**Figure 10:**
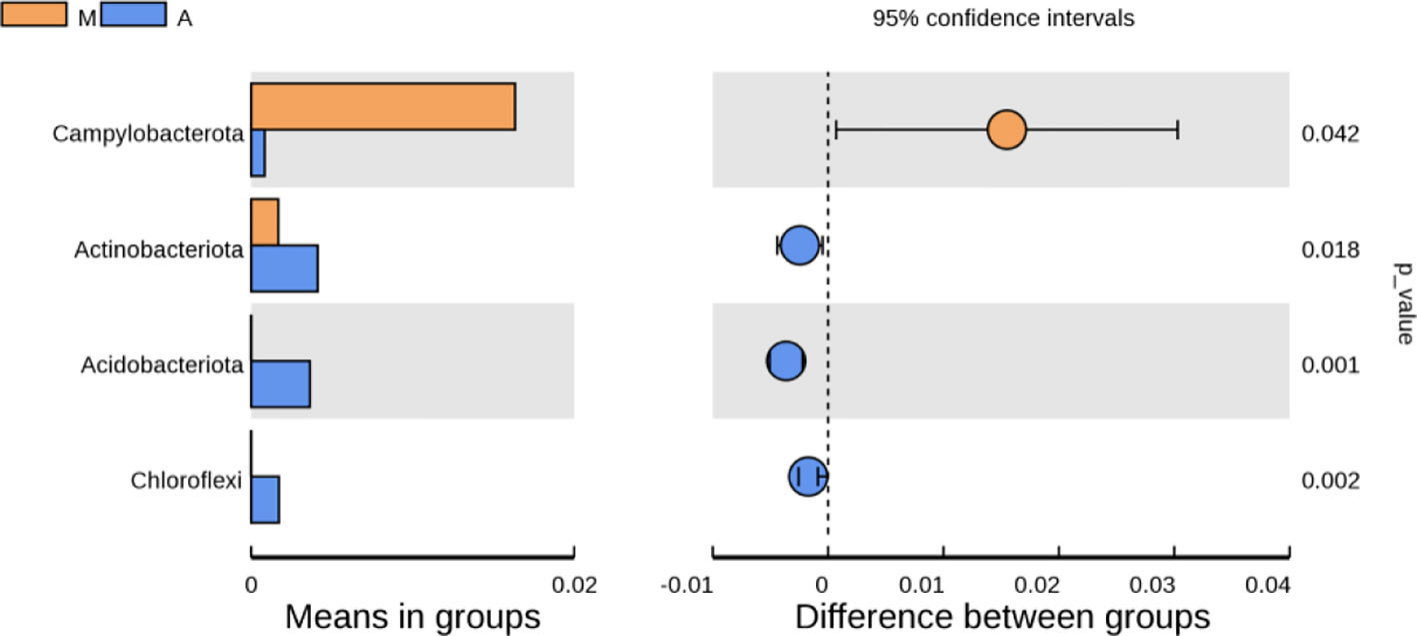
Analysis of Differences in Bacterial microbiota Composition. N, normal group; M, rats treated with 3.5% DSS alone; W: rats treated with DSS plus mesalazine (315 mg/kg); A, rats treated with DSS plus Artemisinin (100 mg/kg).

As shown in **Figure 10**, at the phylum level, compared with the normal group, the model group had an increase in p-Firmicutes and a decrease in p-Bacteroidetes, but there was no statistical significance(*P＞*0.05); p-Tenericutes increased and p-Elusimicrobia decreased (*P＜* 0.05); compared with the model group, there were no significant changes in p-Firmicutes, p-Bacteroidetes, p-Tenericutes, and p-Elusimicrobia in the mesalazine group and artemisinin group (*P＞* 0.05); the abundance of the artemisinin group had an increase in p-Acidobacteria, p-Chloroflexi, Bud p-Gemmatimonadetes, p-Nitrospirae, p-Rokubacteria(*P＜*0.05). Compared with the mesalazine group, there were no significant changes in p-Firmicutes, p-Bacteroidetes, p-Tenericutes, and p-Elusimicrobia in the artemisinin group (*P＞* 0.05). The abundance had an increase in p-Proteobacteria,P-Actinobacteria, p-Acidobacteria, p-Chloroflexi, p-Gemmatimonadetes, p-Nitrospirae, p-Rokubacteria, p-Latescibacteria and p-Armatimonadetes (*P＜*0.05); At the order level, the abundance of Verrucomicrobia in the artemisinin group was significantly higher than that in the model group and mesalazine group (*P＜*0.05).

#### 3.6.6 Beta diversity display

**Figure 11:**
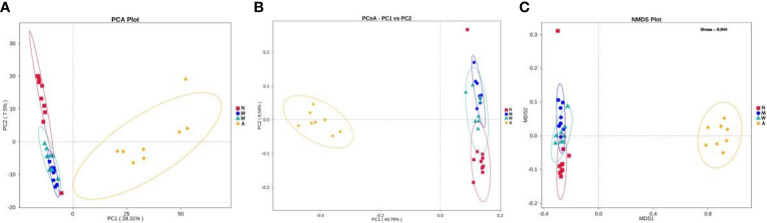
Multiple sample PCA analysis **(A)**. Multiple sample PCoA analysis **(B)**. Multiple sample NMDS analysis **(C)**. N, normal group; M, rats treated with 3.5% DSS alone; W: rats treated with DSS plus mesalazine (315 mg/kg); A, rats treated with DSS plus Artemisinin (100 mg/kg).

The results of PCA principal component analysis and PCoA principal coordinate analysis showed that the microbiota of the mesalazine group had a high similarity with the normal group and the model group, and was quite different from the artemisinin group. The non-metric multidimensional scaling analysis (NMDS) based on β diversity distance reflects the degree of difference between different samples through the distance between points. As shown in **Figures 11A–C** the samples in the normal group, model group and mesalazine group overlap. It showed that the composition of intestinal microbiota between the groups was relatively similar, and the samples in the artemisinin group did not overlap, indicating that the intestinal microbiota was significantly different from the other three groups.

#### 3.6.7 Lefse and functional analysis prediction

**Figure 12:**
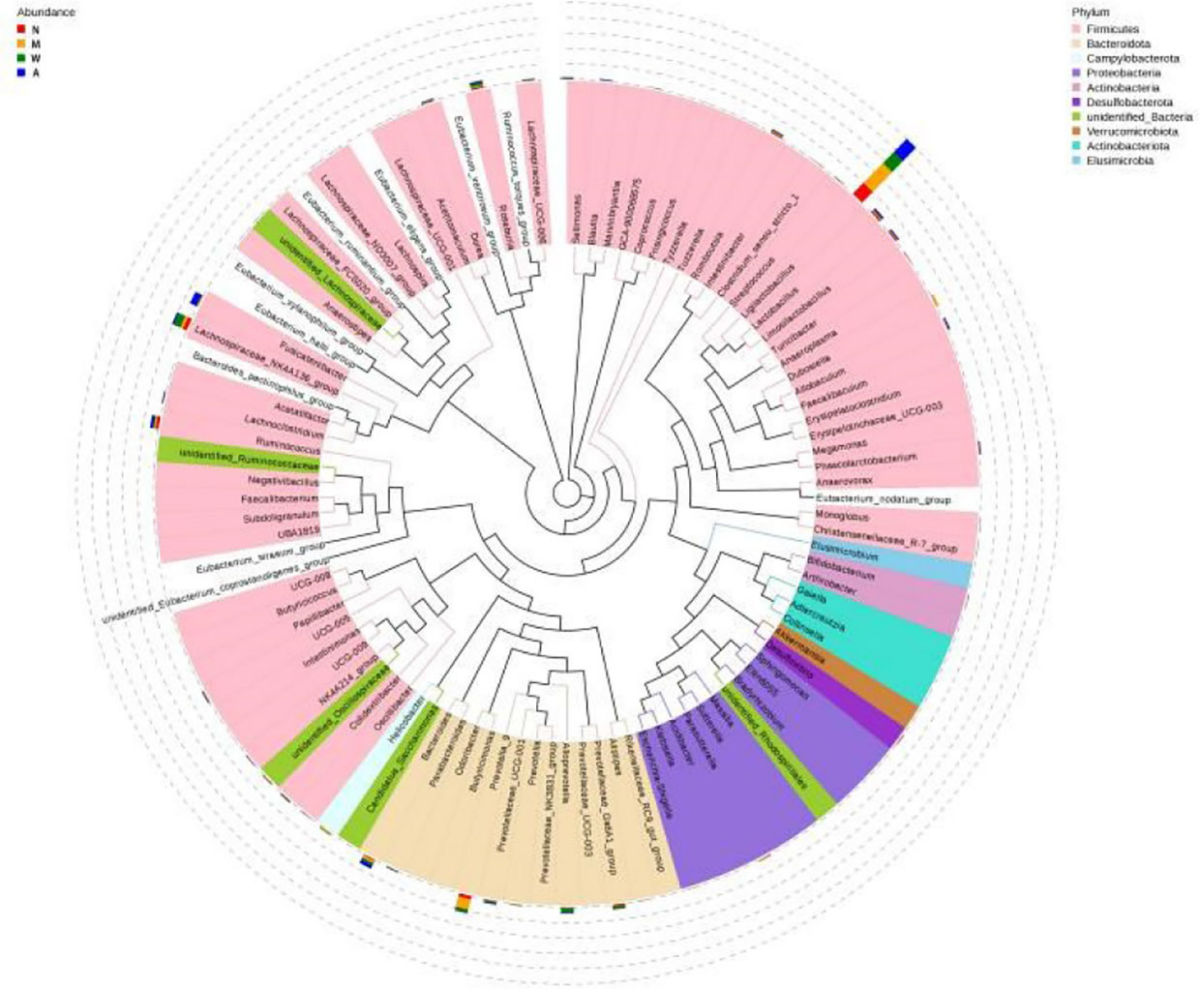
Cladogram. N, normal group; M, rats treated with 3.5% DSS alone; W, rats treated with DSS plus mesalazine (315 mg/kg); A, rats treated with DSS plus Artemisinin (100 mg/kg).

As shown in the **Figure 12**, Ligilactobacillus and Prevotella-9 were the dominant microbiota in the model group, Ligilactobacillus and Lachnospiraceae-NK4A136-group were the dominant microbiota in the mesalazine group, and Ligilactobacillus and Fusicatenibacter were the dominant microbiota in the artemisinin group. The clade map showed that the types of intestinal microbiota of rats did change after artemisinin treatment. The above results indicated that DSS administration and artemisinin treatment had certain effects on the intestinal microecological structure and population.

## 4 Discussion

UC is a common inflammatory bowel disease. Conventional western medicine treatment is mainly based on anti-inflammatory drugs. The research on anti-inflammatory drugs of UC began in laboratory and gradually developed to clinical application. Although it has good clinical efficacy, there are inevitably some side effects. The strong potential of TCM in the treatment of UC has gradually been recognized by the public. More and more evidences (9, 24) indicate that intestinal microbiota imbalance, inflammation and oxidative stress are closely related to its pathogenesis. In recent years, with the deepening of research, artemisinin and its derivatives have been found to have positive therapeutic effects on IBD and colon cancer (25, 26). Therefore, this study applied modern network pharmacology and molecular docking to explore the potential mechanism of artemisinin on UC, and verified the molecular function through animal experiments.

In this study, 55 potential targets of artemisinin and 4810 related targets of UC were identified. The construction of the PPI network reviewed 43 correlated indicators between artemisinin and UC. Ten core goals were further screened out according to the degree value. GO functional analysis showed that the core targets were mainly related to fatty acid metabolic process, response to molecule of bacterial origin, and rhythmic process, etc.KEGG pathway analysis further revealed that the targets were mainly involved in cell metabolism, apoptosis, endocrine, nerve conduction, immune response and other activities. The good results of molecular docking further verifies that artemisinin has a good advantage in the treatment of ulcerative colitis at the molecular level, and also provides an excellent premise for further animal experiments. In addition, animal-based experiments have shown that artemisinin can treat UC by inhibiting inflammation, anti-oxidation and modulating intestinal microbiota.

The experimental results indicated that the pathological changes in the colon of the DSS-induced UC rat model showed inflammatory cell infiltration, goblet cell edema and crypt abscess at the ulcer site. In this study, it was found that artemisinin effectively reduced the infiltration of inflammatory cells, congestion, edema and ulcers. The results indicate that artemisinin can alleviate colon damage of UC. In addition, DAI and CMDI scores are commonly used to evaluate UC (27). According to our experimental results, artemisinin significantly reduced the high DAI and CMDI values induced by DSS, which again shows that artemisinin can effectively alleviate the disease activity in rats. The above results support our further study on the mechanism of artemisinin in the treatment of UC.

The inflammatory state of UC involves the changes of various inflammatory mediators, such as tumor necrosis factor-α (TNF-α), C-reactive protein (CRP), interleukin-6 (IL-6), etc (28). CRP is a biomarker reflecting systemic inflammation and has been evaluated as a marker of IBD activity (29–35). TNF-α has long been considered as a key inflammatory mediator of colonic inflammation (36). Blocking TNF-α activity has been shown to be an effective way to inhibit inflammation. Consistent with previous studies, artemisinin treatment significantly inhibited DSS-induced CRP and TNF-α levels in this study. In addition, in our study, serum CRP and TNF-α levels in rats treated with luteolin were significantly lower than those treated with mesalazine. Network pharmacology analysis showed that artemisinin mainly exerts its effects by regulating PTGS1, ESR1, HSP90AA1, NR3C1, MAPK8, CYP3A4, AHR, ABCB1, AR, NOS3 and other targets. PTGS1, ESR1, MAPK8, and AHR Modern have been studied deeply in modern medicine research. PTGS1 is also known as COX-1, has been pointed out in relevant literatures, that PTGS1 is not only an essential enzyme, but also participates in and aggravates inflammatory reactions. ESR1 is a class of estrogen receptors that bind to estrogen and act. Estrogen may be involved in the development of inflammatory bowel disease, so estrogen receptors may also be involved in inflammation. The protein encoded by MAPK8 is a member of the MAP kinase family, which is involved in cell proliferation, differentiation, Transcriptional regulation, development and other processes as an integration point for various biochemical signals. Various cells modulate immediate early gene expression in response to cell stimulation by stimulating activation and targeting specific transcription factors. TNF-α activates MAP kinase, which induces apoptosis. As a transcription factor, AHR is highly expressed in immune cells and can mediate a series of related responses (37). In colitis and colitis related cancers, uncontrolled intestinal immune response to bacterial antigens leads to the production of large amounts of cytokines and chemokines by activated leukocytes and epithelial cells, accompanied by excessive oxidative stress (38). The production of excessive ROS caused by the intestinal microenvironment disrupts the antioxidant system of the intestinal tract, resulting in oxidative damage of the intestinal tract, triggering pro-inflammatory signals and a variety of cytokines. SOD is one of the antioxidant enzymes that scavenge superoxide radical anion in the body, and MDA is an important marker of lipid peroxidation and damage. T-AOC can reflect the total antioxidant capacity of the body, and its level directly reflects the changes of the antioxidant capacity of the body (39). HIF-1α acts as a transcription factor that can regulates cell adaptation to hypoxia levels and supports intestinal barrier development and function (40). This study showed that the mesalazine group and the artemisinin group could reduce the contents of MDA and HIF-1α in serum to different degrees, increase the contents of T-AOC and SOD, and at the same time improve the pathological damage of colon tissue, while the effect of artemisinin group was better than that of mesalazine group, indicating that artemisinin could increase the anti-inflammatory and antioxidant capacity of UC rats.

Recently, more and more studies have begun to focus on the relationship between intestinal microbiota disorder and UC (41–43). Related studies suggest that intestinal microbiota disorder causes alterations in intestinal epithelial permeability, which leads to intestinal immune disorders and chronic inflammatory processes, ultimately leading to the development of UC (44–46). Traditional Chinese medicine has made some achievements in the treatment of UC with its advantages of multi-target and multi-link modulation. Among them, Huang Lian Detoxification Tang can significantly reduce the alpha diversity of intestinal microbiota in mice and has a wide range of antibacterial effects on pathogenic bacteria (e.g. Escherichia, Odoribacter, Alloprevotella) (9). Berberine not only ameliorated colonic injury in DSS-induced UC rats, but also modulated intestinal microbiota by increasing lactic acid-producing and carbohydrate-hydrolyzing bacteria and reducing conditionally pathogenic bacteria (47). The intestinal microbiota diversity and microbiota structure were significantly improved by the treatment with Baitoum decoction, decreasing the relative abundance of harmful bacteria and increasing the relative abundance of beneficial bacteria (48). Similarly, based on alpha diversity indices of Chao1, Ace, simpson and shannon, this study found that artemisinin was equally effective in increasing the diversity and abundance of intestinal microbiota in rats. Although intestinal microbiota diversity and abundance are closely related to UC, their changes are not directly related to UC development (49). Consistent with this, the intestinal bacterial structure of rats in the artemisinin group did not fit well with the normal rat intestinal microbiota structure from the PCA plot, but it did not affect the relief of clinical symptoms of UC in the artemisinin group rats.

Differences in the microbial composition of the different groups were analyzed at the phylum family level. Compared with the normal group, the model group showed an increase in Firmicutes and a decrease in Bacteroidetes, but there was no statistical significance. After artemisinin treatment, the diversity and abundance of intestinal microbiota in rats increased, including Acidobacteria, Actinobacteria, Chloroflexbacteria, Budmonobacteria, Nitrospira, p-Latescibacteria, Armor Bacteria, Verrucomicrobia and other levels were detected.

The phylum Bacteroidetes is absolutely dominant in the intestinal and have many functions such as enzymatic carbohydrates, participation in polysaccharide metabolism, bile acid and steroids, and maintenance of normal intestinal physiology, etc., which have an important impact on human health (50). Ritchie, L.E. et al. found that the intestinal damage profile of the DSS-induced UC rat model was negatively correlated with Firmicutes, Actinobacteria and Acidobacteria (51). Relevant studies have shown that (52–54) Lactobacillus can produce lactic by fermentating carbohydrates, which helps digestion and absorption, acidifies the intestinal environment, prevents adhesion of harmful bacteria in the intestinal epithelium, stimulates the production of immunoglobulins, and thus enhances host immunity. Our study found that artemisinin can effectively increase the intestinal Lactobacillus in rats and has a positive effect on improving intestinal function. Ren Z et al. showed that some bacteria of Verrucomicrobia play an important role in host immune stimulation and metabolic signal transduction (55). Studies have shown that tumors can cause significant changes in the intestinal microbiota structure of human and animals. For example, the number of Verrucomicrobia bacteria in patients with early stage liver cancer is significantly lower than that in healthy people (56). Overall, the rats in the artemisinin group showed significant improvements in intestinal microbiota richness, diversity and stability, and reduced severity of colonic inflammation compared with the UC model and the rats in the mesalazine group, which is consistent with previous reports (57, 58). The differences in intestinal microbiota richness, diversity and stability between the mesalazine and artemisinin groups also explained the differences in the severity of colonic inflammation in the two groups, and the determination of the specific effective bacteria needs to be further investigated.

## 5 Conclusion

In conclusion, this study shows that artemisinin is effective in alleviating colonic injury and inhibiting inflammatory response in UC rats. In addition, the structure and composition of the intestinal microbiota of UC rats were changed after artemisinin treatment, but the difference of the microbiota was significant, which needs further research and exploration. This study provides a certain research basis for the study of the therapeutic mechanism of UC, and laid a foundation for further research on the mechanism of artemisinin improving UC.

## Data availability statement

The original contributions presented in the study are included in the article/supplementary material. Further inquiries can be directed to the corresponding authors.

## Ethics statement

This experiment was approved by the Ethics Committee and Animal Experiment Committee of Hebei Academy of Traditional Chinese Medicine.

## Author contributions

YG, ZL and NC wrote the manuscript and did the statistical analysis. XJ, JW conducted the experiment. HM and RZ prepared the figures. QY and YC conceived and designed the project. All authors contributed to the article and approved the submitted version.

## Funding

This study was supported by the Scientific Research Project of Hebei Provincial Administration of Traditional Chinese Medicine (NO.2020080, NO.2020083 and NO.2020084).

## Conflict of interest

The authors declare that the research was conducted in the absence of any commercial or financial relationships that could be construed as a potential conflict of interest.

## Publisher’s note

All claims expressed in this article are solely those of the authors and do not necessarily represent those of their affiliated organizations, or those of the publisher, the editors and the reviewers. Any product that may be evaluated in this article, or claim that may be made by its manufacturer, is not guaranteed or endorsed by the publisher.
